# Revolutionising orthopaedic imaging: From 2D radiography and computed tomography to 3D volumetric radiography

**DOI:** 10.1002/jeo2.70161

**Published:** 2025-02-13

**Authors:** Ben Efrima, Amit Benady, Jari Dahmen, Gino M. M. J. Kerkhoffs, Jon Karlsson, Federico Giuseppe Usuelli

**Affiliations:** ^1^ Department of Orthopedics Tel Aviv Medical Center Tel Aviv Israel; ^2^ Levin Center of Surgical Innovation and 3D Printing Tel Aviv Medical Center Tel Aviv Israel; ^3^ Department of Orthopedic Surgery and Sports Medicine Amsterdam Movement Sciences, Amsterdam UMC, Location AMC University of Amsterdam Amsterdam The Netherlands; ^4^ Academic Center for Evidence‐based Sports Medicine (ACES), Amsterdam UMC Amsterdam The Netherlands; ^5^ Amsterdam Collaboration for Health & Safety in Sports (ACHSS) International Olympic Committee (IOC) Research Center, Amsterdam UMC Amsterdam The Netherlands; ^6^ Department of Orthopaedics, Sahlgrenska Academy University of Gothenburg Gothenburg Sweden; ^7^ Ankle and Foot Unit, Humanitas San Pio X Hospital Milan Italy

Since its inception in 1972 [12], spiral computed tomography (CT) has become an invaluable diagnostic tool, offering precise three‐dimensional (3D) image acquisition [3]. However, spiral CT has several potential limitations; it is relatively expensive and space‐consuming, making it accessible only to large clinics or hospitals. Additionally, it requires high radiation exposure, posing potential risks to both patients and medical personnel. Lastly, image acquisition is performed in a non‐weight‐bearing position.

Cone beam computed tomography (CBCT) emerged in the late 1990s as an alternative. CBCT machines utilise X‐rays in the form of a large cone that covers the designated surface to be examined. Unlike the traditional CT, CBCT machines employ rotating flat panel. This design allows the machine to irradiate a large volume area rather than a thin slice, requiring only a single rotation to gather all the necessary information for reconstructing the region of interest (ROI) and creating 3D reconstructions quickly, with low radiographic exposure. Moreover, the scanner is relatively small and affordable compared with spiral CT, making it suitable for in‐office use. Finally, a major disadvantage of CBCT was its high susceptibility to metal artifacts. Currently, computer algorithms are implemented in the devices, as such, it is not any more of a problem than it is for conventional CT [12].

The concept of acquiring images in a vertical position, rather than solely horizontally, originated in dentistry with the clinical introduction of CBCT [12]. Over the last decade, vertical CBCT has been introduced to orthopaedic surgery in the form of weight‐bearing CT (WBCT) [5, 6, 7, 8, 13]. This technology enables image acquisition in the weight‐bearing position, providing a 3D view of the upper and lower limb under the load of the body's weight. The use of WBCT has significantly improved the visualisation of foot and ankle morphology under load‐bearing conditions, enhancing diagnostic capabilities and providing more precise postoperative follow‐up. Consequently, in certain countries, surgeons are using WBCT as an in‐office device, enabling orthopaedic surgeons to provide accurate 3D imaging already during patient visits without the need for referral to large medical centres. This development has made 3D image acquisition more accessible in the ordinary day‐to‐day practice.

## FROM TWO‐DIMENSIONAL SLICES TO AI‐POWERED SEGMENTATION

While WBCT provides a reliable representation of the 3D morphology of the foot, standard imaging software still requires surgeons to rely on 2D slices in the coronal, axial, and sagittal planes within the 3D scan. Traditionally, creating accurate 3D models has required a manual segmentation process, where surgeons had to outline the 3D boundaries of each individual bone. The imaging analysis software would then analyse the manual segmentation to create a 3D model of the scanned area [4, 5, 6, 7]. This segmentation process is time‐consuming and has, therefore, been reserved only for selected cases and large referral centres.

In recent years, the rapid advancement of artificial intelligence (AI) technology has refined and improved this process. Currently, AI‐powered imaging analysis software is capable of semi‐automatic or fully automatic segmentation of accurate 3D models. This development effectively reduces the time‐consuming segmentation process to a matter of minutes, making 3D imaging more widely available. This imaging analysis software includes several valuable complementary features, such as automatic angle measurement algorithms, distance mapping, and virtual osteotomy capabilities, enabling surgeons to plan osteotomies and complex procedures on 3D models and automatically calculate the post‐surgical alignment of the foot and ankle [4, 5, 6, 7, 8]. The integration of WBCT and image analysis software renders the once complex, time‐consuming, and expensive process of 3D image acquisition, segmentation, image analysis, and preoperative planning widely available to orthopaedic surgeons in general.

## PRECISION SURGERY: COULD IT BE AVAILABLE TO ALL?

Achieving precise implementation of preoperative planning still requires advanced surgical capabilities. Currently, the most widely available tools for precision surgery available for orthopaedic surgeons are robot‐assisted surgeries, CBCT‐guided navigation, and patient‐specific instruments [10, 14, 16]. All these methods have been validated and are currently in clinical use. They have been instrumental in improving precision, reducing surgical exposure, and enhancing patient‐reported outcomes compared with standard surgical techniques. Patient‐specific instrumentation has gained immense popularity over the past decade, largely due to advancements in 3D printing technology. This approach enables the custom manufacturing of surgical guides and implants, allowing for patient‐specific surgical solutions [2]. In parallel, computer‐guided navigation leverages three‐dimensional images from cone‐beam CT scans to guide and track surgical instruments or implants in real time, ensuring exceptional precision [9]. Robotic‐assisted surgery allows for precise component placement and alignment using 3D precise bone cutting and soft‐tissue alignment, based on preoperative planning [1]. Much like the original spiral CT, these surgical instruments are expensive and space‐consuming, making them inaccessible to orthopaedic surgeons in general.

In recent years, there has been significant improvement in augmented reality (AR). This exponential improvement is directly correlated to the gradual infiltration of AI capabilities, which will reduce the computer power required for AR. AR goggles are becoming more affordable, and the idea of using AR for precision surgery has captured the imagination of many orthopaedic surgeons. However, to be acceptable for clinical use, AR must overcome three major obstacles: projection, registration, and navigation [11, 15].

In AR‐guided surgery, the model is projected over the surgical site. An overly opaque projection can obscure the surgical site, while an overly transparent projection can make the surgical plan invisible. Therefore, one of the next challenges is to create a well‐balanced projection that allows optimal visualisation of both the surgical site and the plan. The second challenge is registration or aligning the preoperative plan and projection with the actual surgical site. To accurately calibrate and locate the 3D projection, the AR software must be oriented to specific landmarks. Currently, a wide variety of registration tools are available, but they require rigorous validation processes in vitro before proceeding to clinical trials. The final obstacle is navigation, which involves performing the precision surgery. AR goggles can guide surgeons to the initial osteotomy, but once the morphology is altered, the preoperative plan may no longer be accurate. Accurate precision surgery needs to predict all surgical steps.

The rapid introduction of AI capabilities holds the potential to overcome all these obstacles. Accurate, reliable, and effective AR could, in theory, transform precision surgery in the same way that WBCT and AI‐powered image analysis have revolutionised 3D imaging and analysis through decentralised access and diagnostic efficiency. AR could make precision surgery affordable and accessible to every surgeon.

## CONFLICT OF INTEREST STATEMENT

The authors declare no conflict of interest.

## DISCLOSURE

None of the authors have financial or non‐financial interests that are directly or indirectly related to the work submitted for publication except for. Federico G. Usuelli Reports: Relationship with Zimmer Biomet that includes: consulting or advisory and speaking and lecture fees. Relationship with Arthrex Inc that includes: consulting or advisory and speaking and lecture fees. Relationship with Episurf that includes: consulting or advisory and speaking and lecture fees. Relationship with Planmed Oy that includes: consulting or advisory and speaking and lecture fees. Relationship with Geistlich Pharma AG that includes: consulting or advisory and speaking and lecture fees. Relationship with BRM Trust that includes: consulting or advisory and speaking and lecture fees. Relationship with Paragon 28 Inc that includes: consulting or advisory, employment, paid expert testimony and speaking and lecture fees. Membership: International Editor Foot and Ankle International.

## References

[1] Alrajeb R , Zarti M , Shuia Z , Alzobi O , Ahmed G , Elmhiregh A . Robotic‐assisted versus conventional total knee arthroplasty: a systematic review and meta‐analysis of randomized controlled trials. Eur J Orthop Surg Traumatol. 2024;34:1333–1343. 38133653 10.1007/s00590-023-03798-2 PMC10980635

[2] Benady A , Gortzak Y , Sofer S , Ran Y , Rumack N , Elias A , et al. Internal Hemipelvectomy for primary bone sarcomas using intraoperative patient specific instruments‐ the next step in limb salvage concept. BMC Musculoskelet Disord. 2022;23:1012. 36424560 10.1186/s12891-022-05918-1 PMC9685900

[3] Efrima B , Amar E , Rotman D , Elias A , Ejnisman L , Bonin N , et al. Inter and intra‐observer agreement of the 3‐dimensional CT based anterior inferior iliac spine classification system shows fair‐to‐moderate agreement among high volume hip surgeons. Knee Surg Sports Traumatol Arthrosc. 2023;31:50–57. 35648177 10.1007/s00167-022-06999-0

[4] Efrima B , Barbero A , Ovadia JE , Indino C , Maccario C , Usuelli FG . Axial rotation analysis in total ankle arthroplasty using weight‐bearing computer tomography and three‐dimensional modeling. Foot Ankle Surg. 2023;29:506–510. 37193615 10.1016/j.fas.2023.05.001

[5] Efrima B , Barbero A , Ovadia JE , Indino C , Maccario C , Usuelli FG . Classification of the Os calcis subtalar morphology in symptomatic flexible pediatric pes planus deformity using weightbearing CT and distance mapping. Foot Ankle Int. 2023;44:322–329. 36920029 10.1177/10711007231156605

[6] Efrima B , Barbero A , Ovadia JE , Indino C , Maccario C , Usuelli FG . Reliability of cone beam weightbearing computed tomography analysis of total ankle arthroplasty positioning and comparison to weightbearing X‐ray measurements. Foot Ankle Int. 2023;44:637–644. 37231710 10.1177/10711007231173672 PMC10350699

[7] Efrima B , Barbero A , Ramalingam K , Indino C , Maccario C , Usuelli FG . Three‐dimensional distance mapping to identify safe zones for lateral column lengthening. Foot Ankle Int. 2023;44:1061–1069. 37542418 10.1177/10711007231185328

[8] Efrima B , Dahmen J , Barbero A , Benady A , Maccario C , Indino C , et al. Enhancing precision in osteochondral lesions of the talus measurements and improving agreement in surgical decision‐making using weight‐bearing computed tomography and distance mapping. Knee Surg Sports Traumatol Arthrosc. 2024;32:1871–1879. 38591657 10.1002/ksa.12172

[9] Efrima B , Ovadia J , Drukman I , Khoury A , Rath E , Dadia S , et al. Cryo‐surgery for symptomatic extra‐abdominal desmoids. A proof of concept study. J Surg Oncol. 2021;124:627–634. 34043245 10.1002/jso.26528

[10] Lei K , Liu L , Chen X , Feng Q , Yang L , Guo L . Navigation and robotics improved alignment compared with PSI and conventional instrument, while clinical outcomes were similar in TKA: a network meta‐analysis. Knee Surg Sports Traumatol Arthrosc. 2022;30:721–733. 33492410 10.1007/s00167-021-06436-8

[11] Ma L , Huang T , Wang J , Liao H . Visualization, registration and tracking techniques for augmented reality guided surgery: a review. Phys Med Biol. 2023;68:04TR02. 10.1088/1361-6560/acaf23 36580681

[12] Nasseh I , Al‐Rawi W . Cone Beam Computed Tomography. Dent Clin North Am. 2018;62:361–391. 29903556 10.1016/j.cden.2018.03.002

[13] Peiffer M , Dhont T , Cuigniez F , Tampere T , Ashkani‐Esfahani S , D'Hooghe P , et al. Application of external torque enhances the detection of subtle syndesmotic ankle instability in a weight‐bearing CT. Knee Surg Sports Traumatol Arthrosc. 2023;31:4886–4894. 37572141 10.1007/s00167-023-07536-3

[14] Picard F , Deep K , Jenny JY . Current state of the art in total knee arthroplasty computer navigation. Knee Surg Sports Traumatol Arthrosc. 2016;24:3565–3574. 27704159 10.1007/s00167-016-4337-1

[15] Polt M , Viehöfer AF , Casari FA , Imhoff FB , Wirth SH , Zimmermann SM . Conventional vs Augmented Reality‐Guided Lateral Calcaneal Lengthening Simulated in a Foot Bone Model. Foot Ankle Int. 2024;45:773–783. 38501722 10.1177/10711007241237532 PMC11290017

[16] Song EK , Agrawal PR , Kim SK , Seo HY , Seon JK . A randomized controlled clinical and radiological trial about outcomes of navigation‐assisted TKA compared to conventional TKA: long‐term follow‐up. Knee Surg Sports Traumatol Arthrosc. 2016;24:3381–3386. 26831857 10.1007/s00167-016-3996-2

